# Contribution of *Leishmania braziliensis* antigen-specific CD4^+^ T, CD8^+^ T, NK and CD3^+^CD56^+^NKT cells in the immunopathogenesis of cutaneous leishmaniasis patients: Cytotoxic, activation and exhaustion profiles

**DOI:** 10.1371/journal.pone.0229400

**Published:** 2020-03-23

**Authors:** Clarissa F. Cunha, Raquel Ferraz-Nogueira, Vanessa F. A. Costa, Maria Inês F. Pimentel, Thaize Q. Chometon, Marcelo R. Lyra, Armando O. Schubach, Alda Maria Da-Cruz, Alvaro Luiz Bertho

**Affiliations:** 1 Laboratory of Immunoparasitology, Oswaldo Cruz Institute, FIOCRUZ, Rio de Janeiro, Rio de Janeiro, Brazil; 2 Flow Cytometry Core Facility, Oswaldo Cruz Institute, FIOCRUZ, Rio de Janeiro, Rio de Janeiro, Brazil; 3 Laboratory of Surveillance for Leishmaniasis, Evandro Chagas National Institute of Infectology, FIOCRUZ, Rio de Janeiro, Rio de Janeiro, Brazil; 4 Laboratory of Interdisciplinary Medical Research, Oswaldo Cruz Institute, FIOCRUZ, Rio de Janeiro, Rio de Janeiro, Brazil; Universidade de Sao Paulo Instituto de Quimica, BRAZIL

## Abstract

The pathogenesis of cutaneous leishmaniasis (CL) caused by *Leishmania (Viannia) braziliensis* is dictated mainly by the immune-mediated-tissue inflammation developed. The understanding of the immunological mechanisms that generate tissue damage or resolution of lesions is the key to the development of effective vaccine protocols and proper therapeutic schemes. It is clear that the specific immune response mediated by T cells is responsible for the beneficial outcome of the disease, however, the roles of CD4^+^ T, CD8^+^ T, NK and NKT cell subpopulations in immunopathogenesis of CL need to be elucidated. Peripheral blood cells from patients before, during and after the antimonial therapy, as well as healthy individuals (HI) were cultured with (*Lb*AgS) or without (NS) *L*. *braziliensis* antigens (*Lb*Ag). Afterwards, the frequencies of *Lb*Ag-specific-cytotoxic CD8^+^ T, CD4^+^ T, NK and CD3^+^CD56^+^ NKT cells, as well as their activation and exhaustion profiles, were defined by flow cytometry. We observed higher frequencies of CD8^+^ T, NK and CD3^+^CD56^+^ NKT cells and lower frequencies of CD4^+^ T lymphocytes in *Lb*AgS cell cultures from patients before treatment. The specific response to *Lb*Ag resulted in an expansion of cytotoxic-activated CD4^+^ T, CD8^+^ T, and NK cells, before and during treatment, indicating specificity in the response by these cells against *L*. *braziliensis*. Furthermore, comparing the differences of frequencies of cytotoxic-activated CD4^+^T, CD8^+^T, and NK cells, among before and during treatment patients and HI groups, we conclude that these cell populations are in charge of immune response elicited by antimonial therapy. Interestingly, we also observed that NK cells were induced by *Lb*Ag to an exhaustion profile during all clinical stages of the disease. The increased antigen-specific activation and cytotoxic activity are in line with the strong inflammatory response described in this disease, a likely cause of tissue damage. These findings reinforce the involvement of these distinct cytotoxic-activated cell populations in the immunopathogenesis of CL, showing a character of specificity in this immune response.

## Introduction

Cutaneous leishmaniasis (CL) is a group of neglected diseases initiated through the bite of an infected female sand fly vector, with one million cases reported in the last five years worldwide [1]. In Brazil, CL is endemic in several states, including Rio de Janeiro, where is caused mainly by *Leishmania (Viannia)* braziliensis. Clinical characteristics manifest as localized skin lesions that may resolve spontaneously or by antimonial treatment, however may become chronic, leading to severe tissue destruction and disfigurement. The resolution of lesions depends on the magnitude of host innate and adaptive immune responses and the clinical outcome of the disease seems to be due to the T-lymphocytes effector functions and cytokine profiles. Antimonial therapeutic approaches used worldwide are unable to achieve a full-sterile cure and there is no vaccine available yet [2–8].

Much is discussed about the events associated with the infection and its resolution and it has been suggested that CD4^+^ T lymphocytes mediate a protective immunity, whereas CD8^+^ T lymphocytes would have a pathological-cytotoxic role [9–13]. Nevertheless, a protective role for CD8^+^ T cells in the host immune response has already been clearly established [14–18]. It is important to state that these studies have been focused only in classical-cytotoxic-CD8^+^ T cells, however other cell populations as NK, NKT, and even CD4^+^ T cells, with cytotoxic profiles, contribute importantly in the immune response in the CL patients, highlighting the important cytotoxic role performed by CD4^+^ T and NKT cells [19]. Corroborating this statement, we also reported the greatest contribution of CD4^neg^CD8^neg^ T cells and NKT in the cytotoxic events in the CL lesion’s milieu [20]. Due this pluri-cell-populations immune response, it is fundamental to investigate the contributions of these cell populations presenting antigen-specific cytotoxicity (CD107a^+^) [18, 21–23], activation (CD38^+^) [24] and exhaustion (CD279^+^) [25–28] profiles.

For this purpose, we investigated *in vitro Leishmania*-antigen responsiveness of overall and cytotoxic CD4^+^ T, CD8^+^ T, NK and CD3^+^CD56^+^NKT cells and their functional status from peripheral blood of *L*. *braziliensis*-infected CL patients before, during and after antimonial treatment.

## Materials and methods

### Ethical consideration

All participants were volunteers and they signed a written informed consent prior to collection of the blood samples. This study was approved by the National Ethical Clearance Committee of Brazil (CONEP) as well as by the former Evandro Chagas Clinical Research Institute (CEP-IPEC/FIOCRUZ 029/2012, named actually as National Institute of Infectology–INI), Brazil. The ethical principles were in accordance with Declaration of Helsinki on human subject research.

### Subjects

Twenty-three patients from the state of Rio de Janeiro, Brazil, had positive diagnosis for *Leishmania braziliensis* and were distributed in three cohorts: **1)** 15 patients with active disease, evaluated before the beginning of antimonial treatment (group **BT**, 36.71±3.74 years old, 80% males; n = 15); **2)** 15 patients during antimonial treatment, evaluated between the 15^th^ and 20^th^ day after beginning treatment (group **DT**, 40.0±3.65 years old, 73.33% males; n = 15); **3)** 9 patients after the end of treatment, showing recent clinical cure, corresponding to the 80^th^ day after the beginning of treatment (group **CC**, 42.20±4.38 years old, 88.88% males; n = 9). It is important to note that some patients were found repeated in groups, so the number of blood samples processed varied in each group, totalizing 39 blood samples from patients throughout the study. The healthy individuals group (**HI**, 30.29±4.78 years old, 14.20% males; n = 7) was formed of seven healthy individuals recruited from *L*. *braziliensis*-non-endemic areas and was not observed any clinical or epidemiological evidence of the disease.

The CL was diagnosed by clinical, laboratorial and epidemiological criteria and submitted to antimoniate-N-methyl-glucamine treatment, according to the Surveillance Manual of American Cutaneous Leishmaniasis, Brazilian Ministry of Health. Differential diagnosis to other diseases through mycological and bacteriological tests was also performed. The exclusion criteria adopted were individuals younger than 18 and older than 70 years old; presence of comorbidity or pregnancy; previous treatment with anti-*Leishmania* drugs; and recent visit to an endemic area outside Rio de Janeiro state. Furthermore, none of the patients showed progression to mucosal or disseminated clinical forms. After treatment, all patients presented clinical cure, which was characterized by full epithelialization of ulcerated lesions, regression of crusts, desquamation and infiltration.

### Isolation and cryopreservation of the peripheral blood mononuclear cells

The isolation and cryopreservation of samples from patients and HI were performed as described elsewhere [19]. Briefly, peripheral venous blood from CL patients and healthy individuals were collected into heparinized polypropylene tubes (Vacutainer System, BD Biosciences, San Jose, CA, USA), diluted in RPMI-1640 medium supplemented with 10 mM HEPES, 1.5 mM L-glutamine, 0.04 mM 2-mercaptoethanol, antibiotics (200 IU/mL penicillin and 200 mg/mL streptomycin) (all from Sigma-Aldrich, St. Louis, MO, USA) pH 7.2 and then added to a Ficoll-Hypaque (Histopaque 1077; Sigma-Aldrich) gradient. After centrifugation at 400 x *g* for 30 min at 20°C, 30 minutes, without brake, the peripheral blood mononuclear cells (PBMCs) ring was collected, cells were then washed three times and resuspended with RPMI-1640 supplemented medium with 10% of fetal calf serum (FCS–Gibco, Invitrogen, Life Technologies, Rockville, MD, USA) and adjusted to 1x10^7^/mL. Then, PBMCs were suspended in 1 mL of freezing solution [90% inactivated FCS plus 10% dimethyl sulfoxide (DMSO—Sigma-Aldrich)] in cryotubes (Nunc^®^, Kamstrupvej, Roskilde, DNK). Afterwards, the cryotubes were placed in a Mr. Frosty^™^ Freezing Container (Thermo Fisher Scientific, Waltham, MA, USA), stored initially at −80°C for 24 h and then stored in liquid nitrogen.

### *In vitro L*. *braziliensis*-antigen-cell-response assay and CD107a evaluation

For *in vitro* assays, cells were defrosted by placing cryotubes at 37°C-water bath for 3 min, diluted in 20%-FCS-supplemented RPMI-1640 medium and then, aliquots of 20 μL of sample, plus 20 μL of trypan blue (0.5%) reagent were homogenized, and 8 μL were transferred to a Neubauer chamber (Paul Marienfeld GmbH & Co. KG, Lauda-Königshofen, Germany) to count unstained-viable cells using optic microscopy. Samples showing a cell viability inferior than 80% were discarded. Cells were adjusted to 3x10^6^/mL in RPMI medium supplemented with 10% FCS and then 100 μL of cell-containing medium were displaced in each well of a 96-well round-bottomed plate (Nunc^®^). A final volume of 200μL per well was managed adding 100μL of supplemented RPMI medium without FCS. The plates were then incubated for 72 hours at 37°C, in a humidified atmosphere of 5% CO_2_, in the presence or absence of 1x10^7^-promastigotes-equivalent-concentration of *L*. *braziliensis* (*Lb*Ag), which was obtained after disruption of parasite for 10-times-freeze/thaw cycles and a subsequent 5-minute ultrasonication. Non-stimulated (NS) and PHA-stimulated (1μg/well, Sigma-Aldrich) cultures were used as negative and positive controls, respectively. Six hours before the end of incubation time, anti-CD107a (BD Biosciences) was added to the cell cultures and, then after one hour, 6μg/mL of BD Golgi Stop containing monensin (BD Biosciences) was also added to each well, according to the protocols previously described [19].

### Flow cytometry

**Fig 1:**
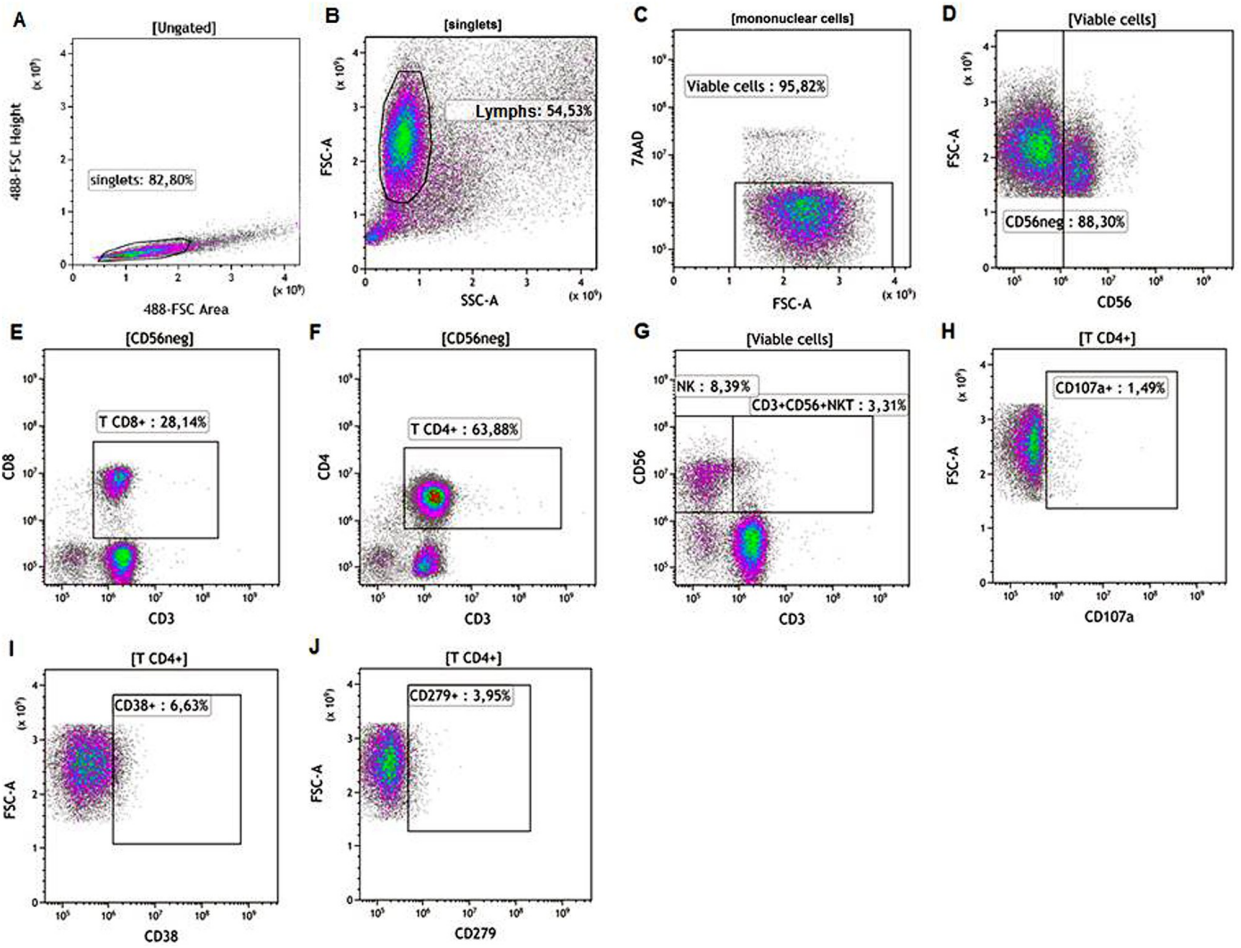
Flow cytometry representative protocol. A gate strategy was performed, as follow: (**A**) to exclude cell aggregates from analyses, cells were gated on **singlets** region in FSC-A *vs*. FSC-H dot-plot; (**B**) a second SSC-A *vs*. FSC-A dot plot was created from **singlets** gate and **Lymphs** region was defined; (**C**) **Viable cells** region were defined by a gate encompassing the 7AAD^neg^ cells in a FSC-A *vs*. 7AAD dot-plot. (**D**) From **Viable cells** gate (7AAD^neg^), **CD56**^**neg**^ cells were defined in CD56 *vs*. FSC-A dot-plot and from **CD56**^**neg**^ gate, **(E)** CD8^+^ T lymphocytes and **(F)** CD4^+^ T lymphocytes were determined; (**G**) CD3 *vs*. CD56 dot-plot, gated on **Viable cells**, was used to define NK and CD3^+^CD56^+^ NKT cells. (**H-J**) From a gate on each cell population (exemplified here by CD4^+^T cells), the frequencies of CD107a^+^, CD38^+^ and CD279^+^ cells were determined in respective dot-plots.

Cells were collected after incubation time, washed in PBS and submitted to flow cytometry staining protocol, consisted by the following monoclonal-antibody panel: anti-CD3, anti-CD4, anti-CD8, anti-CD56, anti-CD38, anti-CD279 (BD Biosciences; Beckman Coulter, Brea, CA, EUA; or BioLegend, San Diego, CA, EUA), including 7-aminoactinomycin D (7AAD, Sigma-Aldrich) for exclusion of dead cells. Cells were incubated for 20 minutes at 4°C in the dark, washed by centrifugation and acquired in flow cytometer within 24 h. At least 50,000 events from each sample were acquired through MoFlo Astrios Cell Sorter flow cytometer (Beckman Coulter). Single stained controls were used to set compensation parameters, while fluorescence-minus-one (FMO) and isotype-matched-antibody controls were used to set analysis regions. After acquisition, flow cytometric analysis to evaluate the frequencies of CD8^+^ T, CD4^+^ T, NK and CD3^+^CD56^+^NKT cells and their expressions of CD107a, CD38 and CD279 were performed using Kaluza Analysis Software (Beckman Coulter). A gate strategy was performed as follow (Fig 1): to exclude cell aggregates from analyses, cells were gated on **singlets** region in FSC-A *vs*. FSC-H dot-plot (Fig 1A); a second SSC-A vs FSC-A dot plot was created from **singlets** gate and lymphocytes region was defined (**Lymphs**; Fig 1B); to exclude dead cells from analyses, **Viable cells** region were defined by a gate encompassing the 7AAD^neg^ cells (unstained-viable cells) in a FSC-A *vs*. 7AAD dot-plot (Fig 1C). From **Viable cells** gate (7AAD^neg^), **CD56**^**neg**^ cells were defined in CD56 *vs*. FSC-A dot-plot (Fig 1D) and from **CD56**^**neg**^ gate, CD8^+^ T lymphocytes and CD4^+^ T lymphocytes were determined by plotting CD8 *vs*. CD3 and CD4 *vs*. CD3 respectively (Fig 1E and 1F). A CD3 *vs*. CD56 dot-plot, gated on **Viable cells**, was used to define NK and CD3^+^CD56^+^ NKT cells (Fig 1G). From a gate on each cell population (exemplified here by CD4^+^T cells), the frequencies of CD107a^+^, CD38^+^ and CD279^+^ cells were determined in respective dot-plots (Fig 1H–1J).

### Statistical analysis

The *Wilcoxon matched-pairs signed rank test* was used for comparison between two related samples, and the *Mann–Whitney U-test* was used for comparison between each two groups for unpaired observations. A probability level of P≤0.05 was considered statistically significant. Statistical analysis was performed with GraphPad Prism version 6.0 for Windows (GraphPad Prism 6 Software, San Diego, CA, USA).

## Results

### Patients’ characteristics

The Table 1 summarizes some clinical and epidemiological data of the patients enrolled in this study. The mean age of 24 CL patients was 37 ± 14.26 (± SEM) years and 79% of them (n = 19) were of male sex. The number of lesions ranged from one to five, with a diameter ranging from 14 to 130 mm, considering the diameter of the largest lesion. The duration of the illness was determined in days and considered the period comprising the beginning of the appearance of the lesion reported by the patient until the moment of its introduction in the study (day 01), which varied from 15 to 330 days. Montenegro skin test (MST) was positive in all patients who were submitted to the test and it was not performed in the HI group.

**Table 1:** Characteristics of cutaneous leishmaniasis patients enrolled in this study.

		Patient ID	Age (years)	Gender	Number of lessions	Duration of illness (days)	Lesion size (mm)	MST^d^ (mm)
**Patients**	BT (before treatment) n = 15	**1**	22	M^a^	1	40	34	24
		**2**	56	F^b^	1	60	36	36
		**3**	24	M	1	90	45	n.d.^c^
		**4**	28	M	1	30	20	11
		**5**	44	M	1	120	28	n.d.
		**6**	29	M	1	120	50	n.d.
		**7**	70	M	1	15	-	25
		**8**	46	M	4	180	75	27
		**9**	52	M	1	90	24	47
		**10**	51	F	1	60	40	n.d.
		**11**	32	F	1	120	130	n.d.
		**12**	30	M	5	45	-	20
		**13**	52	M	1	120	22	n.d.
		**14**	31	M	1	330	47	n.d.
		**15**	59	M	1	120	60	11
	DT (during treatment) n = 15	**2**	56	F	1	60	36	36
		**3**	24	M	1	90	45	n.d.
		**18**	19	M	1	60	22	18
		**6**	29	M	1	120	50	n.d.
		**19**	26	M	4	60	-	n.d.
		**20**	47	M	1	30	45	14
		**7**	70	M	1	15	-	25
		**8**	46	M	4	180	75	27
		**9**	52	M	1	90	24	47
		**10**	51	F	1	60	40	n.d.
		**21**	19	M	1	30	30	22
		**11**	32	F	1	120	130	n.d.
		**12**	30	M	5	45	-	20
		**15**	59	M	1	120	60	11
		**16**	23	F	1	120	35	n.d.
	CC (clinical cure) n = 9	**3**	24	M	1	90	45	n.d.
		**6**	29	M	1	120	50	n.d.
		**19**	26	M	4	60	-	n.d.
		**20**	47	M	1	30	45	14
		**22**	30	M	4	120	-	n.d.
		**23**	25	M	1	-	14	n.d.
		**24**	42	F	1	90	35	10
		**21**	19	M	1	30	30	22
		**15**	59	M	1	120	60	11

^a^M–male;

^b^F–female;

^c^n.d.- not done;

^d^MST—Montenegro skin test

### *Stimulation with L*. *braziliensis* antigens leads to a contraction of CD4^+^ T lymphocytes and an expansion of CD8^+^ T, NK and CD3^+^CD56^+^NKT cells in cell cultures from CL patients

**Fig 2:**
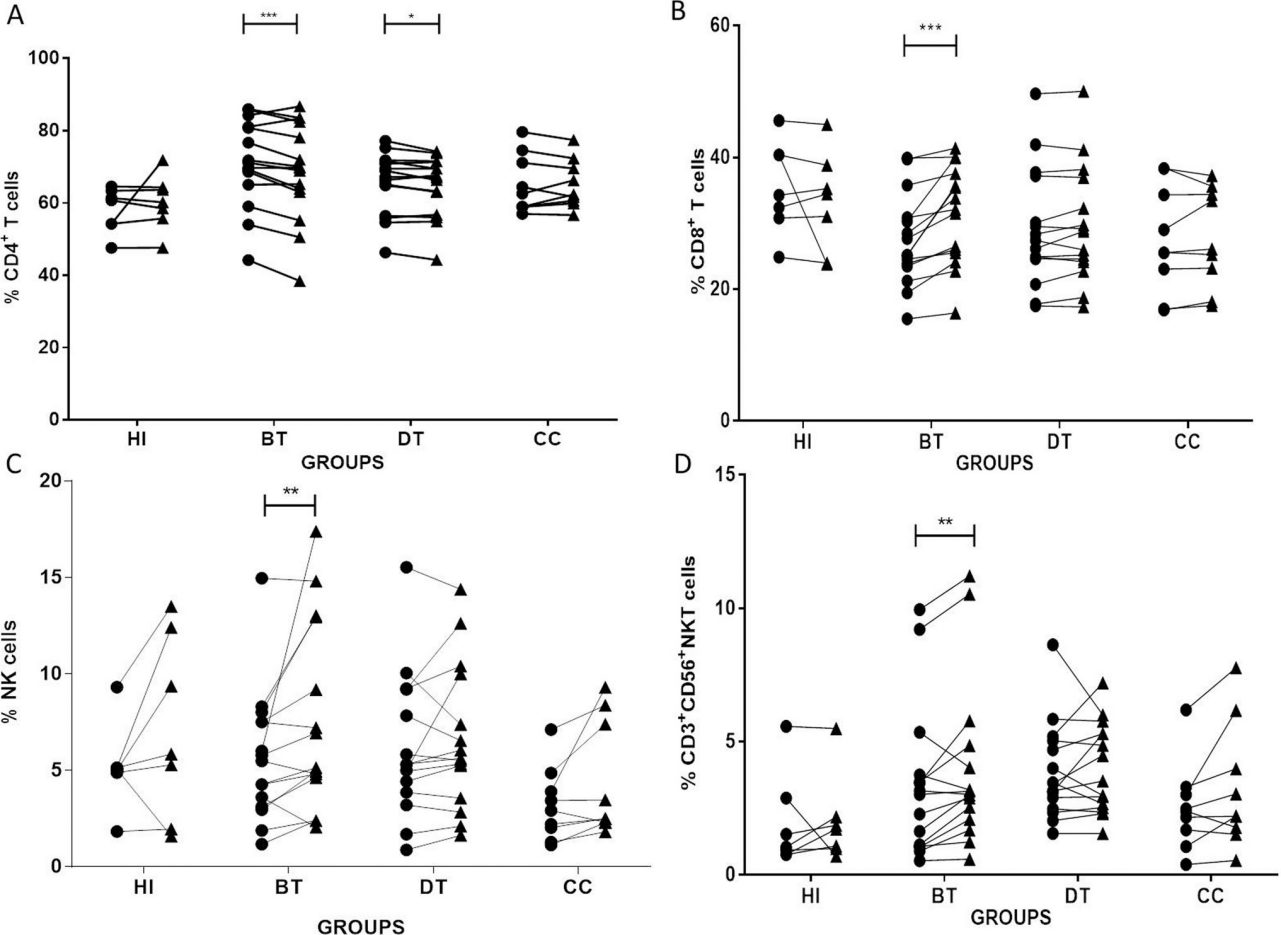
Analysis of CD4^+^T, CD8^+^T, NK and CD3^+^CD56^+^NKT cells in *L*. *braziliensis*-antigens-stimulated-cell cultures. Paired–analyses graphics show frequencies of (**A**) CD4^+^ T; (**B**) CD8^+^ T; (**C**) NK; and (**D**) CD3^+^CD56^+^ NKT cells from before treatment (BT; n = 15); during treatment (DT; n = 15); clinical cure (CC; n = 9) patients. HI (n = 7) represents cell cultures from healthy individuals. Solid lines connect the results of non-stimulated (NS) (●*)* and stimulated with *L*. *braziliensis* antigens (*Lb*AgS) **(**▲) cell cultures for the same individual. Statistical analyses were performed by nonparametric Wilcoxon matched pairs test. Results between NS (●*)* and *Lb*AgS **(**▲) cell cultures were considered significant with P≤0.05. *(P≤0.05); **(P≤0.01); ***(P≤0.001).

Our first question was whether stimulation with *L*. *braziliensis* antigens (*Lb*Ag) induced modulation in the frequencies of CD8^+^ T, CD4^+^ T, NK and CD3^+^CD56^+^ NKT cells. We observed an *Lb*Ag-specific-reduction of frequencies of CD4^+^ T cells in *Lb*AgS cell cultures from BT group (mean±SEM: 68.74±3.50) compared to NS cell cultures (72.39±3.28) (P<0.001); and in *Lb*AgS cell cultures from DT group (mean±SEM: 64.61±2.16) compared to NS cell cultures (65.46±2.21) (P<0.05). (Fig 2A). In opposite, we observed expansions of CD8^+^ T, NK and CD3^+^CD56^+^ NKT cells in the *Lb*AgS cell cultures (31.25±1.91; 7.51±1.25; and 3.96±0.79, respectively) compared to NS cell cultures [27.41±1.81 (P≤0.01); 5.65±0.87(P≤0.01); and 3.32±0.74 (P≤0.01)] only from BT patients (Fig 2B–2D). HI showed no significant changes in the frequencies of overall cell populations comparing NS and *Lb*AgS cell cultures (Fig 2A–2D).

### Evaluation of CD38 expression by *Lb*Ag-stimulated cells

**Fig 3:**
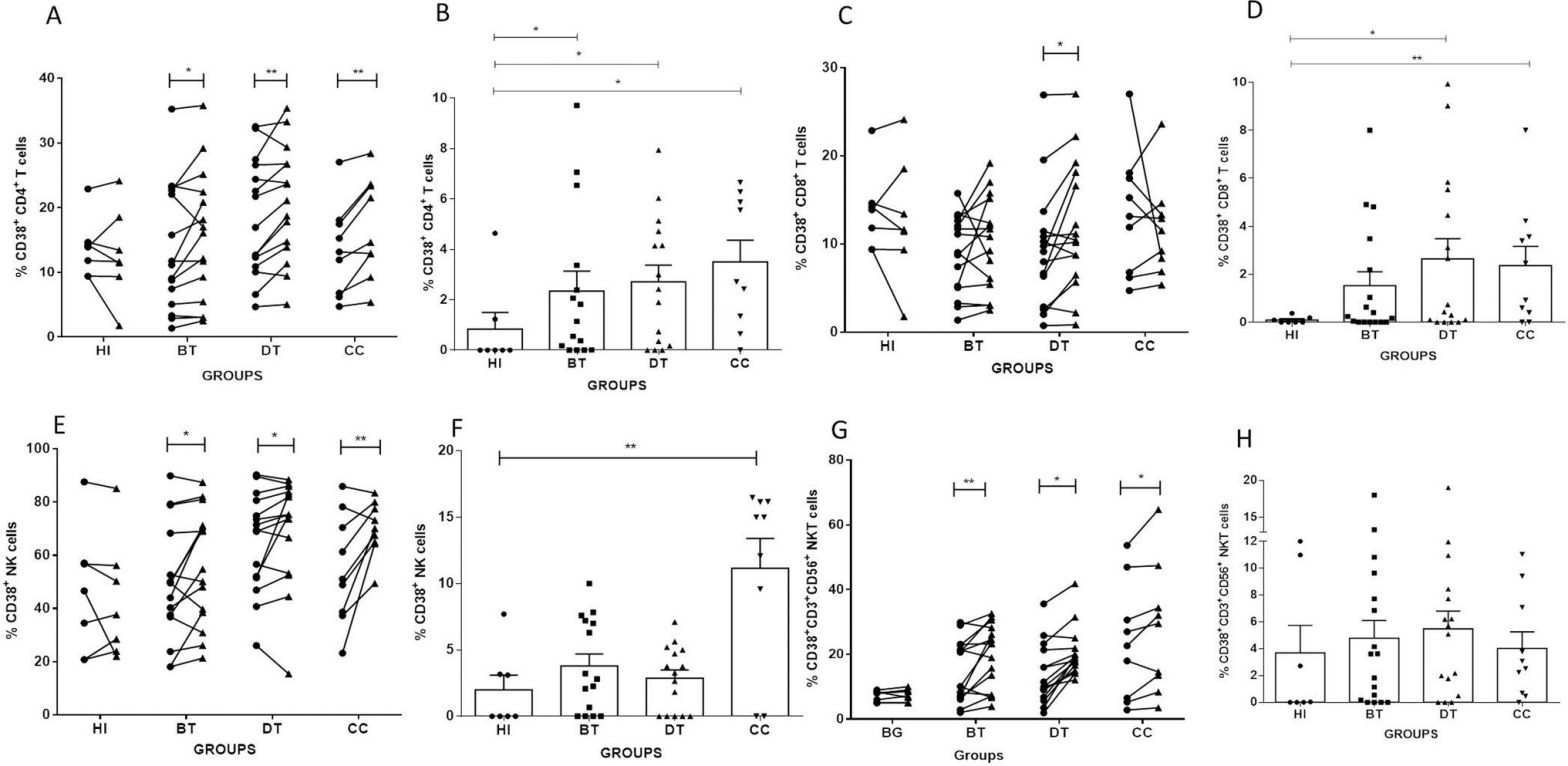
Analyses of frequencies of CD38^+^CD4^+^T, CD38^+^CD8^+^T, CD38^+^NK and CD38^+^CD3^+^CD56^+^NKT cells from cell cultures stimulated with *L*. *braziliensis* antigens. Paired–analyses graphics show frequencies of (**A**) CD38^+^CD4^+^ T; (C) CD38^+^CD8^+^ T; (E) CD38^+^NK; and (G) CD38^+^CD3^+^CD56^+^ NKT cells from before treatment (BT; n = 15); during treatment (DT; n = 15); clinical cure (CC; n = 9) patients. HI (n = 7) represents cell cultures from healthy individuals. Solid lines connect the results of non-stimulated (NS) (●*)* and stimulated with *L*. *braziliensis* antigens (*Lb*AgS) **(**▲) cell cultures for the same individual. Statistical analyses were performed by nonparametric Wilcoxon matched pairs test. Results between NS (●*)* and *Lb*AgS **(**▲) cell cultures were considered significant with P≤0.05. *(P≤0.05); **(P≤0.01). Column graphs **B**, **D**, **F** and **H** represent the mean±SEM of percentages of (**B**) CD38^+^CD4^+^ T; (**D**) CD38^+^CD8^+^ T; (**F**) CD38^+^ NK; and (**H**) CD38^+^CD3^+^CD56^+^ NKT cells stimulated with *L*. *braziliensis* antigens (*Lb*AgS) minus non-stimulated (NS) cell percentages from BT (n = 15); DT (n = 15); CC (n = 9) and HI (n = 7) groups. Statistical analyses were performed by nonparametric Mann-Whitney test. Results were considered significant with P≤0.05 comparing to HI. *(P≤0.05); **(P≤0.01).

We evaluated by paired analysis the frequencies of CD4^+^ T; CD8^+^ T; NK and CD3^+^CD56^+^ NKT cells expressing CD38 in NS and *Lb*AgS cell cultures from BT, DT, CC patients and HI. We observed significant expansions of CD38^+^CD4^+^ T, CD38^+^ NK and CD38^+^CD3^+^CD56^+^ NKT cells in *Lb*AgS cell cultures from BT, DT and CC groups, comparing to NS cell cultures (Fig 3A, 3E and 3G). While evaluating the frequencies of CD38^+^CD8^+^ T cells in *Lb*AgS cell cultures, we observed expansions in these cells only in DT patients (Fig 3C). It is important to note that HI showed no frequency variations when analyzing overall CD38^+^ cell populations (Fig 3A, 3C, 3E and 3G).

Evaluating cell frequencies of *Lb*AgS minus NS cell cultures, we observed higher frequencies of CD38^+^CD4^+^T and CD38^+^CD8^+^T lymphocytes in cell cultures from DT and CC patients (2.71±0.61 and 2.63±0.84; 3.37±0.78 and 2.35±0.80, respectively) compared to cell cultures from HI (0.83±0.65 and 0.09±0.05, P≤0.05 and P≤0.01; respectively) (Fig 3B and 3D). We also observed a higher percentage of CD38^+^ NK cells in the cell cultures from CC comparing to HI groups (11.16±2.23 and 1.99±1.1, respectively; P≤0.01) (Fig 3F). It was not observed differences in CD3^+^CD56^+^ NKT cells among all groups studied (Fig 3H).

### *L*. *braziliensis* antigens increase the frequencies of overall CD107a^+^-cell populations in CL patients

**Fig 4:**
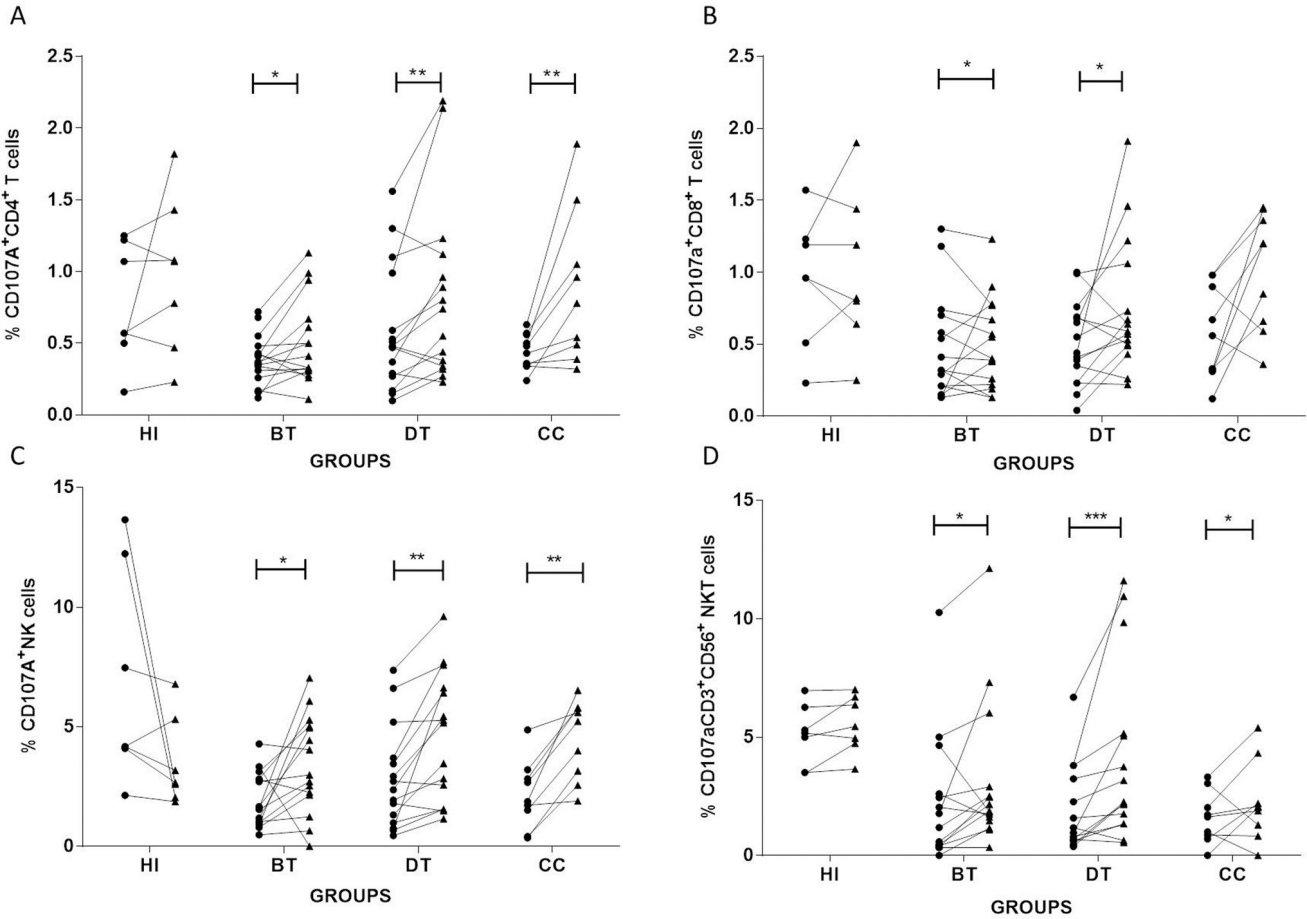
Analyses of frequencies of CD4^+^ T, CD8^+^ T, NK and CD3^+^CD56^+^NKT cells expressing CD107a in the presence or lack of *L*. *braziliensis* antigens. Paired–analyses graphics show frequencies of (**A**) CD4^+^ T; (**B**) CD8^+^ T; (**C**) NK; and (**D**) CD3^+^CD56^+^ NKT cells from before treatment (BT; n = 15); during treatment (DT; n = 15); clinical cure (CC; n = 9) patients. HI (n = 7) represents cell cultures from healthy individuals. Solid lines connect the results of non-stimulated (NS) (●*)* and stimulated with *L*. *braziliensis* antigens *Lb*AgS **(**▲) cell cultures for the same individual. Statistical analyses were performed by nonparametric Wilcoxon matched pairs test. Results between NS (●*)* and *Lb*AgS **(**▲) cell cultures were considered significant with P≤0.05. *(P≤0.05); **(P≤0.01); ***(P≤0.001).

To determine changes in the frequency of CD107a^+^-cytotoxic cells involved in a *Leishmania*-specific response, we evaluated the frequencies of CD4^+^ T; CD8^+^ T; NK and CD3^+^CD56^+^ NKT cells expressing CD107a in NS and *Lb*AgS cell cultures from BT, DT and CC patients and HI. We observed expansions of CD107a^+^CD4^+^ T, CD107a^+^NK and CD107a^+^CD3^+^CD56^+^ NKT cells in *Lb*AgS cell cultures in all patient groups (Fig 4A, 4C and 4D). While evaluating CD107a^+^CD8^+^ T cells in *Lb*AgS cell cultures, we observed expansion in DT and CC groups and no variations BT patients (Fig 4B). It is important to note that HI showed no changes in frequencies of CD107a^+^CD8 T cells in response to *Lb*Ag (Fig 4A–4D).

### *L*. *braziliensis*-antigens induce an increase of cytotoxic-activated CD4^+^ T, CD8^+^ T and NK cell frequencies in BT and DT patients

**Fig 5:**
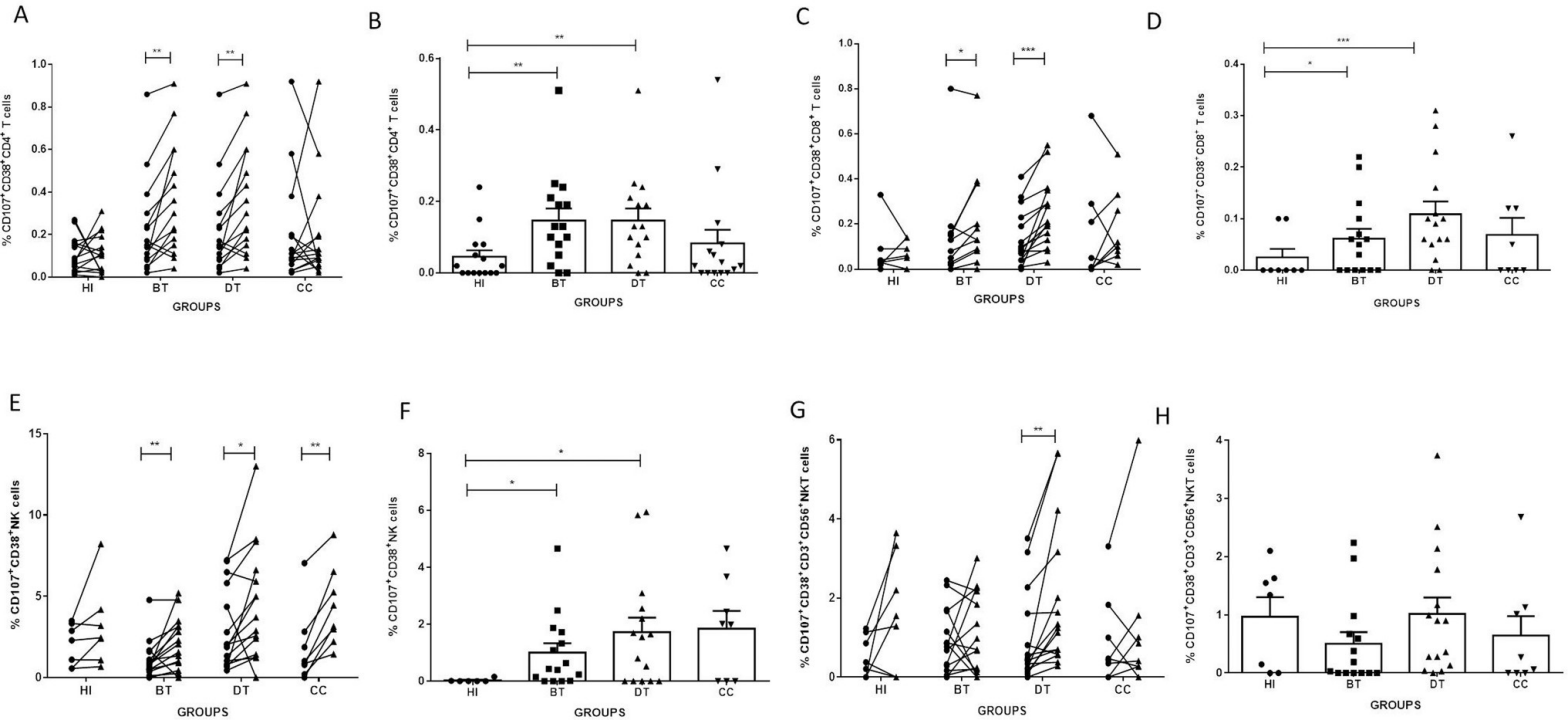
Analyses of frequencies of CD107a^+^CD38^+^CD4^+^ T, CD107a^+^CD38^+^CD8^+^ T, CD107a^+^CD38^+^ NK and CD107a^+^CD38^+^CD3^+^CD56^+^ NKT cells in cell cultures stimulated with *L*. *braziliensis* antigens. Paired–analyses graphics show frequencies of (**A**) CD107a^+^CD38^+^CD4^+^ T; (C) CD107a^+^CD38^+^CD8^+^ T; (E) CD107a^+^CD38^+^NK; and (G) CD107a^+^CD38^+^CD3^+^CD56^+^ NKT cells from before treatment (BT; n = 15); during treatment (DT; n = 15); clinical cure (CC; n = 9) patients. HI (n = 7) represents cell cultures from healthy individuals. Solid lines connect the results of non-stimulated (NS) (●*)* and stimulated with *L*. *braziliensis* antigens (*Lb*AgS) **(**▲) cell cultures for the same individual. Statistical analyses were performed by nonparametric Wilcoxon matched pairs test. Results between NS (●*)* and *Lb*AgS **(**▲) cell cultures were considered significant with P≤0.05. *(P≤0.05); **(P≤0.01); ***(P≤0.001). Column graphs **B**, **D**, **F** and **H** represent the mean±SEM of frequencies of (**B**) CD107a^+^CD38^+^CD4^+^ T; (**D**) CD107a^+^CD38^+^CD8^+^ T; (**F**) CD107a^+^CD38^+^ NK; and (**H**) CD107a^+^CD38^+^CD3^+^CD56^+^ NKT cells in *Lb*Ag-stimulated (*Lb*AgS) minus non-stimulated (NS) cell cultures from BT (n = 15); DT (n = 15); CC (n = 9) and HI (n = 7) groups. Statistical analyses were performed by nonparametric Mann-Whitney test. Results were considered significant with P≤0.05 comparing to HI. *(P≤0.05); **(P≤0.01); ***(P≤0.001).

We evaluated by paired analysis the frequencies of CD4^+^ T; CD8^+^ T; NK and CD3^+^CD56^+^ NKT cells expressing simultaneously CD107a and CD38 in NS and *Lb*AgS cell cultures from BT, DT and CC patients and HI. We observed significant expansions of CD107a^+^CD38^+^CD4^+^ T, CD107a^+^CD38^+^CD8^+^ T and CD107a^+^CD38^+^ NK cells in *Lb*AgS cell cultures from BT and DT patient groups comparing to NS cell cultures (Fig 5A, 5C and 5E). Moreover, we observed significant expansions of CD107a^+^CD38^+^ NK cells in *Lb*AgS cell cultures from CC group (Fig 5E). While evaluating the frequencies of CD107a^+^CD38^+^CD3^+^CD56^+^ NKT cells in *Lb*AgS cell cultures, we detected expansions in these cells only in DT patients (Fig 5G). It is important to note that HI showed no variations in frequencies when analyzing the overall CD107a^+^CD38^+^ cell populations (Fig 5A, 5C, 5E and 5G).

Then, we performed analyses subtracting cell frequencies detected in *Lb*AgS minus NS cell cultures and we observed significant differences in CD107a^+^CD38^+^CD4^+^ T, CD107a^+^CD38^+^CD8^+^ T and CD107a^+^CD38^+^ NK cells in cell cultures from BT and DT patients (0.15±0.03, 0.15±0.03 and 1.00±0.33; 0.06± 0.01, 0.10±0.02 and 1.72±0.50, respectively) compared to cell cultures from HI (0.04±001, 0.02±0.01 and 0.02±0.03, respectively) (Fig 5B, 5D and 5F). No differences in CD107a^+^CD38^+^CD3^+^CD56^+^ NKT cells were observed among the groups studied (Fig 5H).

### *L*. *braziliensis* antigens induce NK exhaustion in CL patients

**Fig 6:**
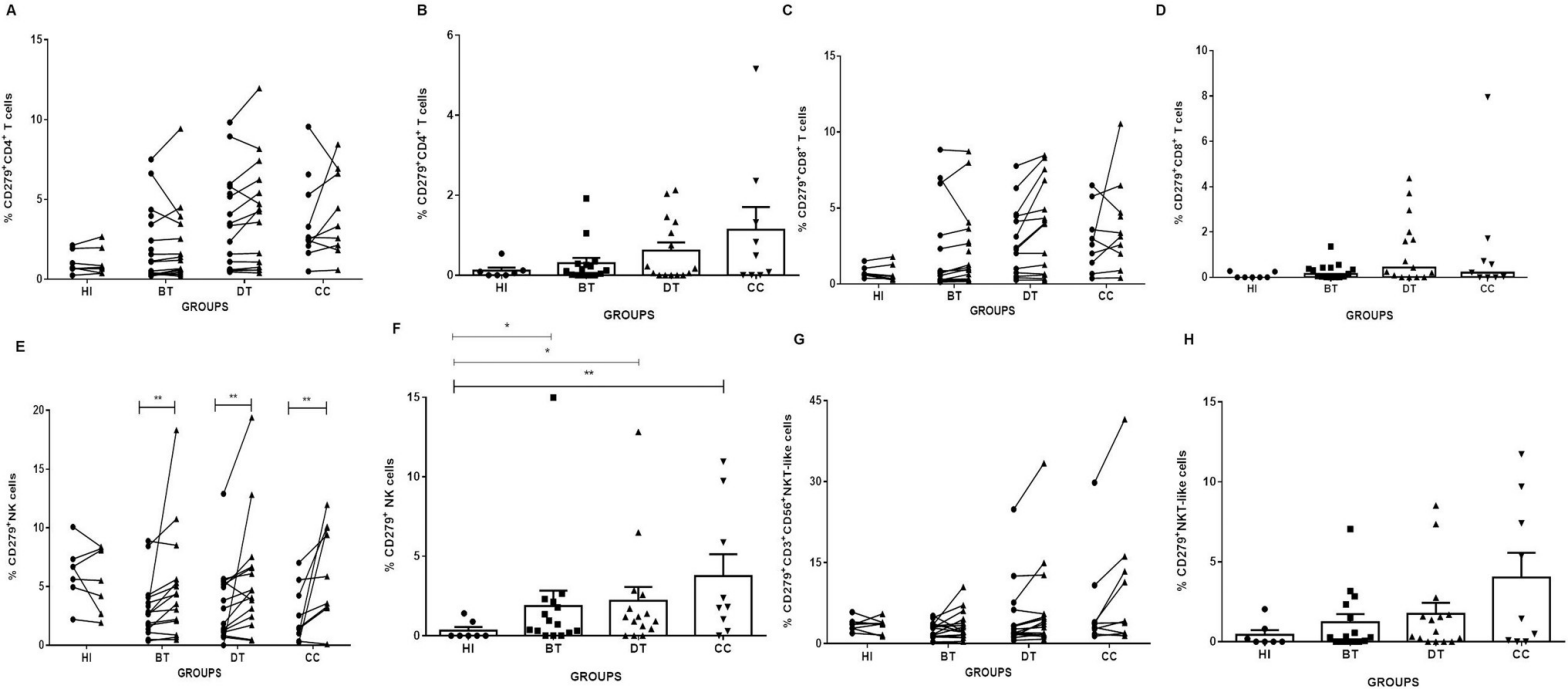
*L*. *braziliensis* antigens induce increase CD279^+^ NK cells in cell cultures from BT, DT and CC patients. Paired–analyses graphics show frequencies of (**A**) CD279^+^CD4^+^ T; (C) CD279^+^CD8^+^ T; (E) CD279^+^NK; and (G) CD279^+^CD3^+^CD56^+^ NKT cells from before treatment (BT; n = 15); during treatment (DT; n = 15); clinical cure (CC; n = 9) patients. HI (n = 7) represents cell cultures from healthy individuals. Solid lines connect the results of non-stimulated (NS) (●*)* and stimulated with *L*. *braziliensis* antigens (*Lb*AgS) **(**▲) cell cultures for the same individual. Statistical analyses were performed by nonparametric Wilcoxon matched pairs test. Results between NS (●*)* and *Lb*AgS **(**▲) cell cultures were considered significant with P≤0.05. *(P≤0.05); **(P≤0.01). Column graphs **B**, **D**, **F** and **H** represent the mean±SEM of frequencies of (B) CD279^+^CD4^+^ T; (D) CD279^+^CD8^+^ T; (F) CD279^+^NK; and (H) CD279^+^CD3^+^CD56^+^ NKT in *Lb*Ag-stimulated (*Lb*AgS) minus non-stimulated (NS) cell cultures from BT (n = 15); DT (n = 15); CC (n = 9) and HI (n = 7) groups. Statistical analyses were performed by nonparametric Mann-Whitney test. Results were considered significant with P≤0.05 comparing to HI. *(P≤0.05); **(P≤0.01).

The evaluation of CD279 expression is related to cell exhaustion and was conducted in this study by comparing frequencies of CD4^+^ T, CD8^+^ T, NK and CD3^+^CD56^+^ NKT cells expressing CD279 in NS and *Lb*AgS cell cultures from BT, DT, CC and HI groups. We observed that *Lb*Ag induces high frequencies of CD279^+^ NK cells from BT, DT and CC patients (6.17±1.7; 6.06±1.26; 7.33±1.55, respectively) comparing to NS cell cultures (4.22±1.27; 4.34±1.1; 3.42±1.05, respectively; P≤0.01) (Fig 6E). Furthermore, subtracting cell frequencies of *Lb*AgS minus NS cell cultures we observed significant differences between frequencies of CD279^+^ NK cells in cell cultures from BT, DT and CC patients (2.20±0.86, P≤0.05 and 3.75±1.37; P≤0.01, respectively) comparing with cell cultures from HI (0.32±0.21) (Fig 6F). The other cell populations were not statistically different in either *Lb*AgS to NS cell cultures paired comparisons or in frequency analysis (Fig 6A–6D, 6G and 6H).

## Discussion

The immunopathogenesis of human CL is dictated largely by the type and magnitude of the host´s innate and adaptive immune response, but also by differences between the infecting *Leishmania spp*. A protective role performed by CD4^+^ and CD8^+^ T lymphocytes in the immune response against *Leishmania braziliensis*, with defined cytokine network, has been exhaustively reported [12–17]. Nevertheless, in some situations, a strong inflammatory response contributes to lesion progress and to exacerbation of the disease, which has been associated with cytotoxic events [9–13]. Recently, our group investigated the contributions of cytotoxic CD4^+^ T, CD8^+^ T, NK and NKT cells in the immune response in blood from CL patients before, during and after antimonial treatment, highlighting an important cytotoxic activity by CD4^+^ T and NKT cells, differently from classical-cytotoxic cells, as CD8^+^ T and NK cells [19]. Corroborating this statement, we also reported the greatest contribution of CD4^neg^CD8^neg^ T cells and NKT in the cytotoxic events in the CL lesion’s milieu [20].

Occurred to us the importance to investigate if these cytotoxic-cell contributions have an antigen-specific-response profiles. In the current study, we observed that *Lb*Ag induced a contraction in CD4^+^ T lymphocyte frequencies in BT and DT patients and an expansion in CD8^+^ T, NK and CD3^+^CD56^+^ NKT cell frequencies from BT patients. These findings corroborate our previous report, which showed lower frequencies of CD4^+^ T cells in BT patients, being predisposed to reestablishment with therapy and recovery to normal levels in CC patients, suggesting that these cells would take part in the CL healing process [19]. Despite this noted contraction of CD4^+^ T lymphocytes, the functionality of these cells does not appear to be impaired, since we observed an increase of CD38^+^CD4^+^ T cells in *Lb*AgS cell cultures from BT and DT patients, indicating an activation profile of CD4^+^ T cells in patients presenting infection. Moreover, we observed specific expansions of cytotoxic-CD107a^+^CD4^+^ T cells in *Lb*AgS cell cultures from all patient groups, suggesting an *Lb*Ag-specific response of cytotoxic-CD107a^+^CD4^+^ T cells, mainly during the antimonial therapy phases, may resulting in the clinical cure of patients. This statement is in consonant with others authors who reported that cured CL patients from endemic areas for *L*. *major* exhibited increased frequencies of cytotoxic-CD4^+^ T lymphocytes in response to *L*. *major* antigen [29]. In addition, evaluating the expression of CD279 –molecule related to cellular exhaustion—we observed that *Lb*Ag did not have influence in the CD279^+^CD4^+^ T-cell frequencies, indicating an effector-functional status of this cell subset.

It is not fully understood how CD8^+^ T cells get activated in leishmaniasis, but it is clear that they do respond during *Leishmania* infection [23]. In our study, we observed an expansion of CD8^+^ T lymphocytes in *Lb*AgS cell cultures from BT patients. Interestingly, our results clear showed an expansion of frequencies of cytotoxic CD8^+^ T cells in *Lb*AgS cell cultures from DT and CC patient groups, indicating that *Lb*Ag-specific-cytotoxic CD8^+^ T cells might be related to an efficient treatment protocol. Furthermore, we verified that these cytotoxic CD8^+^ T cells were in a high activation status in *Lb*AgS cultures from DT patients and the frequencies of activated-cytotoxic CD8^+^ T cells are higher in DT and BT patients comparing to HI. This observation strongly suggests that *Leishmania* antigens induce the increase of activated-cytotoxic CD8^+^ T cells. It is important to note that CD8^+^ T cells did not show an exhaustion profile at any clinical stage studied, which denotes that these cells maintain their effector functional status.

NK cells are important effector lymphocytes of the innate immune system, playing critical roles in antitumor and anti-infection defenses [30]. Several findings indicate a protective role of NK cells in human leishmaniasis, including the enhanced proliferation of NK cells within PBMC from cured or control individuals residing in an endemic area as compared to patients with active CL lesions, after *in vitro* stimulation with *L*. *aethiopica* [31]; and the influx of NK cells into lesions of CL patients who showed a response to treatment [32]. There are few reports about the role of NK cells in CL caused by *L*. *braziliensis*. Some authors have proposed that NK cell-mediated cytotoxicity contributes to tissue damage in *L*. *braziliensis* infection [30]. Reinforcing these findings, we observed in the present study a positive modulation of CD38 + CD107 + NK cells in cell cultures of BT and DT patients, that is, with active disease, even with therapy already started. In our previous *ex vivo* study, we detected low frequencies of cytotoxic-NK cells in CL patients, which was associated with a possible suppression of NK cell activity by the parasite [18], phenomenon also noted in *L*. *major* infection [33]. In this sense, assessing the frequencies of cells in exhaustion (CD279^+^), we observed an increase of NK cell frequencies in *Lb*AgS cell cultures in all patient groups when compared with NS cultures, suggesting that NK exhaustion profile might be associated with antigenic specificity. These findings are in agreement with others that correlated the exhaustion status with antigen dependency and persistence, as well as inflammatory process that occur in CL lesions [34–37].

The role of NKT cells in the resistance or susceptibility towards *Leishmania* infections remains to be more elucidated. It was already reported that the activation of NKT cells in *Leishmania* infection is resulted by a direct pathway where *Leishmania* antigens are presented by a CD1d bind to invariant TCR and then acting both in producing cytokines and with cytotoxic activity, lysing target cells [38, 39]. In the current investigation we observed that *Lb*Ag induces CD3^+^CD56^+^NKT cell activation, leading to an expansion of cytotoxic-CD107a^+^CD3^+^CD56^+^ NKT in all CL patient groups. Previous report of our group showed high percentages of total and cytotoxic-CD107a^+^NKT cells in the lesions of CL patients, reinforcing the involvement of NKT cells with cytotoxic activity in CL [20]. These findings together strengthen the important role CD3^+^CD56^+^NKT cells in the immune response established by the host during antimonial therapy, contributing to the resolution of disease [19, 20].

Taking all results together we may conclude that *Lb*Ag-specific-cytotoxic response in CL is developed by different cell populations, highlighting the role of non-classical cytotoxic cells, as CD4^+^T and NKT cells.

In the current study, the increased frequencies when cytotoxic cells were stimulated with *Leishmania* antigens strongly indicate that CD4 and NKT cell populations would play an important role in disease resolution as they present these high frequencies in patients during therapy and clinically. In this context, cytotoxic CD8^+^ T cells showed high levels of activation during the antimonial therapy, indicating a key contribution of these cells in the clinical resolution of CL.

Regarding cytotoxic NK cells, despite presenting a considerable level of activation, they presented with high frequencies of exhaustion cells during treatment and clinical cure, indicating that in the presence of *Leishmania* antigens there is a down modulation of these cells, leading to the cure of the disease. Since cell exhaustion status is characterized by poor effector function or senescence, where cells lose their ability to produce cytokines and degranulation, preventing them for performing their optimal capability, we may strongly suggest that NK cells would be related to a harmful cytotoxic action in the pathogenesis of human cutaneous leishmaniasis.
